# Commentary on: Public Perception of Ideal Breast Shape

**DOI:** 10.1093/asjof/ojab055

**Published:** 2021-12-13

**Authors:** Sai L Pinni, Marissa M Tenenbaum

**Affiliations:** Washington University School of Medicine, St. Louis, MO, USA; Plastic and Reconstructive Surgery, Washington University School of Medicine, Barnes Jewish Hospital, St. Louis, MO, USA

Thank you to the authors for their work “Public Perception of Ideal Breast Shape.” ^1^ In this article, the authors used crowdsourcing to determine the general public’s preferences for breast aesthetics following breast augmentation. Although aesthetic preferences for breast surgery have been previously studied in the literature, this comprehensive analysis adds great value to this ever-growing body of research. Moreover, the fluctuating and malleable nature of aesthetic preferences compels constant inquiry regardless.

Crowdsourcing through Amazon Mechanical Turk (mTurk) (Amazon Web Services, Amazon, Seattle, WA) has become a widely accepted and reliable methodology of assessing the opinions and attitudes of the general public across various medical and surgical specialties.^2,3^ One of the most notable features of crowdsourcing is the ability to survey large populations in a very short time frame, demonstrating great utility. This approach is a shift from the more tedious methods previously used to survey the general public. For example, a 2016 study from Lewin et al surveyed 2000 Swedes through regular mail to obtain aesthetic preferences for nipple-areolar complex (NAC) position.^4^ Their 813 respondents answered 4 total questions: 2 on demographics (age and gender) and 2 on NAC aesthetics to indicate preferred position, 1 for the frontal view and 1 for the lateral view. In comparison, this article’s authors received 960 complete responses after recruiting 1000 mTurk workers or Turkers to answer 19 questions and obtained preferences for a total of 10 characteristics of breast aesthetics across both frontal and lateral views.

Images were adapted from the 2019 article from Lee et al., “An Ideal Female Breast Shape in Balance with the Body Proportions of Asians.” All images depict the same skin tone and body habitus, which appear to be a Fitzpatrick skin type I or II and a relatively low body mass index (BMI). Future studies with variation in skin tone and body habitus will be essential for revealing any potential differences in breast shape preferences across these factors.

Demographics collected in this study included age group, gender, ethnicity, marital status, number of children, number of children in household, education level, and socioeconomic status (SES). Most respondents were white (52%), married (65%), and men (60%) between the ages of 25 and 34 years old (49%), and this age bracket represents the average ages of patients undergoing breast augmentation across multiple geographical populations.^5^ Though the authors mentioned that Turkers are representative of the US internet population, respondents were not limited to the United States. While most surveys ask for annual household income, the authors collected SES subjectively using the MacArthur scale, likely because Turkers were not limited to one country. The utility of this information was unclear as results stratified by subjective SES were not reported. In addition, a 2014 study comparing a US-based mTurk worker sample to the US population also found that Turkers were younger, less likely to earn more than $75,000 US, and more likely to be white or Asian.^6^ Though the SES distribution of Turkers may not reflect that of patients undergoing breast augmentation, understanding the perspectives of populations that may be receiving care at relatively lower rates is crucial for increasing accessibility.

The full racial and ethnic breakdown of the study’s survey respondents was 2% Asian, 8% Black/African, 6% Hispanic/Latino, 24% Indian subcontinent, 7% other/multiracial, and 52% white/Caucasian. Though the representation of certain racial and ethnic demographics was relatively low, the authors believed that sample sizes were adequate for analysis and showed preferences specific to certain groups. Further research into preferences by race/ethnicity may also be useful in making aesthetic surgery more available and tailored to these demographics.

Overall, the general public was not indifferent about breast aesthetics, and respondents had the strongest opinions about nipple directions. The front-facing nipple had the highest ranking, whereas the 20° downward-facing nipple had the lowest ranking. This preference presents a critical opportunity for patient education. Surgeons may recognize that nipple direction may change postoperatively as the skin stretches due to descension of the breast tissue. For this reason, preoperative counseling is required when patient preferences may not be aligned with immediate postoperative results and patients cannot readily appreciate expected changes over time. The authors also acknowledged this concept of balancing patient preference with surgical expertise in terms of large breast implants and the need for tissue expansion. Thus, crowdsourced data present an opportunity to identify potential disconnects between patients and providers that can be bridged with appropriate patient counseling and educational tools.

Data also revealed differences in preferences for breast aesthetics by gender, with men having stronger opinions for ratio preferences for breast width to upper buttock width and lower pole height to breast width. The authors recognized that presenting male and female preferences may be of interest to patients depending on their individual motivations. These gender-stratified preferences also further highlight the importance of preoperative shared decision-making as surgeons may have different preferences from their patients. Previous literature has reported that plastic surgeons have significant differences in breast shape preferences across age, sex, country of residence, and practice type but not ethnic background.^7^ Thus, outcomes may vary by surgeon demographics if patient preferences are not appropriately solicited and incorporated into surgical planning. Since Turkers were not limited to one country, it may have been interesting to see if Turkers also have differences by country of residence, and future studies could determine whether these preferences match those of plastic surgeons in the same country.

In conclusion, this article elucidates the general public’s ideal female breast with the greatest preference for front nipple direction, a breast projection proportion of approximately 1.0, a moderately convex upper breast slope, and a lateral breast width to upper buttock width ratio of 105%. The use of crowdsourcing to obtain this data demonstrates the utility of this methodology in quickly learning aesthetic preferences and the potential for addressing gaps between future patients and providers to improve preoperative counseling. Ultimately, individual patient preferences will matter the most, but this information could play a role in limiting surgeon bias for shared decision-making and surgical goal setting. We commend the authors on their contribution to the growing literature on breast aesthetics.
